# The Association between a 24-Hour Blood Pressure Pattern and Circadian Change in Plasma Aldosterone Concentration for Patients with Aldosterone-Producing Adenoma

**DOI:** 10.1155/2019/4828402

**Published:** 2019-07-29

**Authors:** Hai Li, Jianbin Liu, Juan Liu, Liehua Liu, Minmin Huang, Guohong Wei, Wanping Deng, Zhimin Huang, Xiaopei Cao, Haipeng Xiao, Yanbing Li

**Affiliations:** ^1^Department of Endocrinology and Diabetes Center, The First Affiliated Hospital of Sun Yat-sen University, Guangzhou, Guangdong 510080, China; ^2^Department of Medicine, Eastern Health, Box Hill, VIC 3128, Australia; ^3^Centre for Eye Research Australia, University of Melbourne, Parkville, VIC 3001, Australia

## Abstract

The absence of nocturnal blood pressure (BP) decline is associated with hypertensive complications. Data regarding circadian BP patterns in patients with aldosterone-producing adenoma (APA) are limited and equivocal. We evaluated the circadian BP profile in patients with APA and its relationship with the circadian aldosterone rhythm. BP in patients with APA and in those with essential hypertension (EH) were assessed through in-hospital 24-h ambulatory blood pressure monitoring. Over a 24-h in-hospital period, plasma aldosterone levels taken at midnight, 0400, 0800, 1200, 1600, and 2000 h were measured. To evaluate a correlation between BP and hormone rhythm, we included 27 patients with APA (APA group) and 27 patients with EH (EH group). Both groups had similar age, sex ratio, body mass index, duration of hypertension, family history of hypertension, and lipid profiles. The day-night BP differences in both patient groups were similar, whether expressed as absolute values or percentages. The proportions of patients with dipping BP profiles were also comparable (APA group, 5 of 27; EH group, 7 of 27; *χ*2 = 0.429; P = 0.513). At each time point, APA group plasma aldosterone concentrations (PACs) were higher than those of the EH group. A circadian change in relation to PAC was observed in both groups. A correlation between PAC and BP was statistically nonsignificant in most study patients in either group. Our data indicated that the circadian BP pattern was not associated with a change in PAC levels in patients with APA.

## 1. Introduction

Observational studies have shown a 10%–20% decline in blood pressure (BP) levels during sleep at night in most normotensive individuals and in patients with essential hypertension [1, 2]. The term “dipper” refers to individuals with normal nocturnal fall of blood pressure (BP) and “nondipper” refers to those whose BP does not fall nocturnally. A nondipping BP pattern is associated with hypertensive complications [3].

Data regarding circadian BP patterns in patients with aldosterone-producing adenoma (APA) are limited and equivocal. Previous studies have reported that a circadian BP decline was blunted in APA patients [4, 5], which might contribute to increased cardiovascular disease risk in this population.

It is generally known that, in aldosteronism, overproduction of aldosterone leads to increased sodium and water retention and subsequent hypertension via its genomic effect, which is a relatively long-term action. Recent studies [6] have revealed rapid effects of aldosterone on vascular tone; thus, raising the possibility that aldosterone might play a role in short-term BP regulation.

For patients with APA, it remains unclear whether an increase of plasma aldosterone concentration (PAC) or its change might affect the circadian BP pattern. This study aimed to evaluate the circadian BP profile in patients with APA and its relationship with PAC.

## 2. Methods and Materials

### 2.1. Patients

The study participants comprised patients who had been consecutively referred to our center from June 2011 to March 2013 because of suspected primary aldosteronism (PA) or to exclude secondary hypertension (refractory hypertension [uncontrolled hypertension despite the use of at least 5 different classes of antihypertensive agents], spontaneous or drug-induced hypokalemia, onset in youth [<40 years old], or adrenal incidentaloma). The protocol and informed consent documents were approved by the research ethics board of Sun Yat-sen University. All patients provided their written informed consent.

### 2.2. Preparation of Assessments

Before and during the biochemical evaluation, phenoxybenzamine was administered to control BP, if necessary. Spironolactone or amiloride was discontinued at least 6 weeks prior to evaluation, as were angiotensin receptor blockers, angiotensin converting enzyme inhibitors, or thiazide diuretics at least 4 weeks prior to evaluation. Daily natrium and potassium supplements (160 mEq and 60 mEq, respectively) were administered during the assessment period.

### 2.3. Diagnosis of Primary Aldosterone and Aldosterone-Producing Adenoma

We followed study methods previously described by Yanbing Li et al. in 2017 [7], and all patients underwent a standard diagnostic procedure including repeated measurements of serum potassium, and 24-h urinary aldosterone, plasma aldosterone, and renin activity. For those with elevated urinary aldosterone levels, elevated serum aldosterone levels, or elevated aldosterone-renin ratios, upright-furosemide loading tests [8] were performed as confirmatory tests. A diagnosis of PA was confirmed if plasma renin activity (PRA) levels were <2 ng/ml/h after challenge.

An adrenal spiral computed tomography scan with sections at 0.3 mm intervals was conducted. Once an adrenal lesion was located, adrenalectomy was recommended. The final diagnosis of APA was later confirmed with pathology tests plus correction of hypokalemia postoperatively.

### 2.4. Ambulatory Blood Pressure Monitoring and Simultaneous/Synchronous Hormone Assessment

All in-patients received continuous noninvasive 24-h ambulatory blood pressure monitoring (ABPM) (TM 2430, Higashi-Ikebukuro, Toshima-ku, Tokyo) during hospitalization, following the same standard operating procedures as ambulatory blood pressure monitoring for out-patients. The monitor recorded systolic and diastolic BP every 30 min during the daytime (0700 to 2300 h) and every 60 min through the night (2300 to 0700 h). The ambulatory data were included in the analysis if the monitoring period was >20 h and there were no periods of >2 h without measurements. Dippers or a dipping BP pattern were defined as >10% decline in both systolic and diastolic BP [9, 10].

Within 24 hours of in-patient ABPM, blood samples were drawn at midnight, 0400, 0800, 1200, 1600, and 2000 h, for measurements of plasma aldosterone.

### 2.5. Exclusion of Secondary Hypertension due to Other Common Causes

Urine tests, measurements of serum creatinine, 24-h urine free cortisol, 24-h urine vanillylmandelic acid, and ultrasonography of renal arteries and veins were performed to screen common secondary causes of hypertension. Patients with renal hypertension, pheochromocytoma, or Cushing's syndrome were excluded. If the above tests including 24-h urinary aldosterone, plasma aldosterone, renin activity and aldosterone-renin ratios, 24-h urine free cortisol, 24-h urine vanillylmandelic acid, 24-h urine free cortisol, and 24-hour urine vanillylmandelic acid were normal, a diagnosis of nonfunctional adrenal tumor would be made.

### 2.6. Statistical Analysis

All statistical analyses were performed using SPSS 13.0. Values are expressed as mean ± SD, unless otherwise noted. Values between groups were compared using a two-tailed t-test. Variables that were not normally distributed were log transformed before analyses. Categorical values were compared using a chi-squared test. The correlation between PAC and BP was evaluated using a Pearson's test for each patient. Logistic analysis was performed to evaluate the independent predictors of dipping BP patterns in both groups. Statistical significance was considered at a value of P < 0.05.

## 3. Results

### 3.1. Demographic, Clinical, and Biochemical Profiles of the Study Participants

Forty patients were recruited, of whom 13 were excluded for other causes of secondary hypertension. Twenty-seven patients with APA (APA group) were included and received biochemical and in-patient ABPM assessments, as did 27 patients with EH (EH group). No participants received any antihypertensive medication during the evaluation.

Demographic, biochemical, and hormone values of the two groups are shown in Table 1. The two groups had similar age, sex ratio, body mass index, duration of hypertension, family history of hypertension, and lipid profiles. The patients with APA had lower serum potassium concentration and plasma renin activity, and higher PAC, aldosterone-renin ratio, and 24-h aldosterone secretion, compared to patients with EH.

### 3.2. Blood Pressure Measurements

BP profiles of the two groups are presented in Table 2. The day-night BP differences in patients with APA were similar to those in patients with EH, whether expressed as an absolute value or a percentage. Proportions of patients with dipping BP were also comparable between the two groups (APA group, 5 of 27 patients vs. EH group, 7 of 27; *χ*^2^ = 0.429; P = 0.513).

### 3.3. Circadian Change of Plasma Aldosterone Concentration

**Figure 1:**
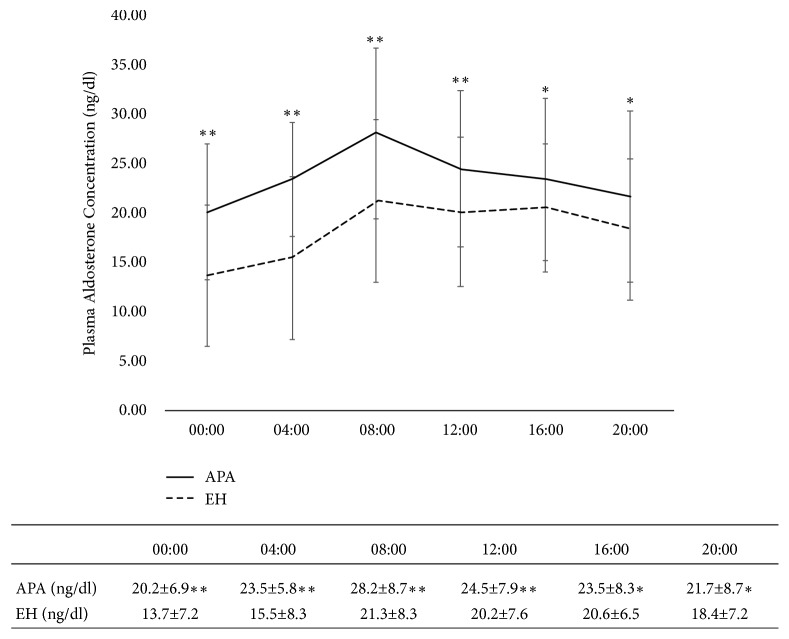
*The circadian change in relation to plasma aldosterone concentration* vs. that in the EH group. *∗*P < 0.05; *∗∗*P < 0.01. Note: the solid line refers to patients with APA, while the dashed line refers to patients with EH. APA, aldosterone-producing adenoma; EH, essential hypertension.

The circadian profiles of PAC in the two groups of patients are shown in Figure 1. The two groups had a similar trend over 24 h, with a peak at 0800 h and a nadir at midnight in each group; however, the EH group had much lower levels at night. The PAC of the APA patients at each time point was higher than that of the EH patients.

### 3.4. Circadian Variation of Blood Pressure and Plasma Aldosterone Concentration

**Figure 2:**
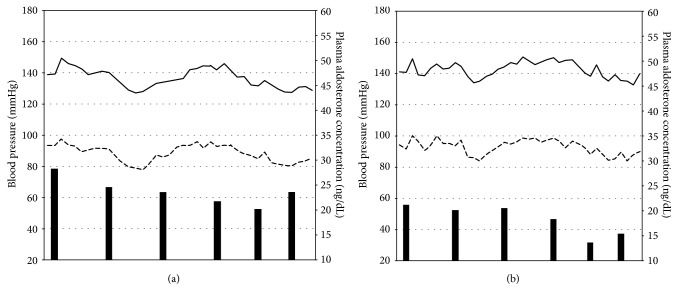
*Circadian variation of blood pressure and plasma aldosterone concentration in patients with aldosterone-producing adenoma (a) and patients with essential hypertension (b).* Note: the solid line represents systolic blood pressure, the dashed line represented diastolic blood pressure, and the bars represent plasma aldosterone concentration.

The circadian change of BP and concomitant PAC in each group are shown in Figure 2. Considering that aldosterone might have a delayed effect on vascular tone (6), we performed linear correlation of PAC with BP recorded at two time points: (1) the time of blood sampling for aldosterone measurement and (2) 1 h after blood sampling. No statistical significance was found (Table 3).

### 3.5. Pearson's Correlation of Plasma Aldosterone Concentration and Blood Pressure

**Figure 3:**
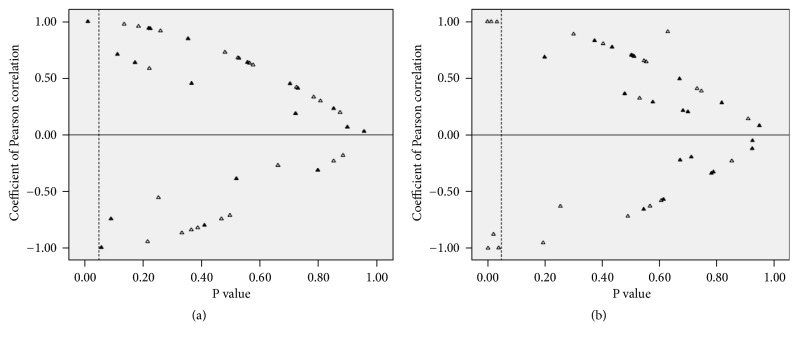
*Pearson correlation of plasma aldosterone concentration and blood pressure ((a) systolic, (b) diastolic) for each patient*. The x-axis represents the P-value yielded according to Pearson's correlations. The dotted line indicates P = 0.05. Most P-values were higher than 0.05 (distributed to the right of the dotted line). Note: each triangle represents a specific patient. A hollow triangle represents a patient with EH and a solid triangle represents a patient with APA.

To ensure a more precise and detailed evaluation, a Pearson's correlation of BP and PAC was conducted for each patient, and the results are presented in Figure 3. The correlation was nonsignificant in most of the studied patients. Logistic regression did not find any independent predictor for dipper BP profiles in either group.

## 4. Discussion

In the present study, we did not find any significant differences in BP level, nocturnal BP decline, or the proportion of patients with a dipping BP pattern, between patients with APA and those with EH, which is consistent with some previous reports [4, 5, 11–15]. However, other studies have shown differing results. Rabbia et al. found significantly fewer dippers among patients with APA in comparison to patients with EH [16]. Less amplitude in the nocturnal fall of BP was also observed in patients with APA [16, 17]. Of note, in the abovementioned studies and in the present study, the absolute proportion of dippers among patients with APA varied significantly, from 18.5% to 91.7%. Thus, based on current data, we cannot yet draw any unequivocal conclusions concerning the BP pattern in patients with APA. When comparing these results, factors such as sample size, medication, daily activity, and sodium intake should be considered.

Previous studies have shown that, in APA patients, the circadian aldosterone change was similar to that of normotensive individuals and those with EH [18–21]. Similarly, in the present study, the diurnal rhythm of plasma aldosterone was present in both APA and EH patients, with a peak at 0800 h and a nadir at midnight (Figure 1). Pathologic production of aldosterone due to APA seemed only to increase PAC but did not modify its circadian rhythm.

The main finding in our study was the absence of statistically significant correlations between PAC and concomitant SBP and DBP (Table 3; Figure 3) in either patients with APA or patients with EH. Furthermore, to assess a delayed effect, linear correlation between aldosterone and BP values was recorded 1 h after the blood sample collection was also performed and still yielded nonsignificant results (Table 3). Therefore, it is likely that aldosterone imposed a minimal instant/short-term (hourly) effect on BP regulation, or that such effect, if any, was so weak that it could be easily confounded due to other mechanisms. Of note, findings from previous studies have been inconsistent. Using a relatively small sample (n = 5), Nicholls et al. [21] reported there was no significant relationship between the plasma aldosterone level and concomitant arterial BP in patients with PA. Imai et al. [11] speculated that aldosterone mediated the BP rhythm, based on their finding that the circadian rhythm of BP and PAC appeared to be synchronous.

A rapid direct vascular effect of aldosterone has recently been reported [6]. In an in vivo study on human forearm vascular reactivity, Romagni et al. [22] reported a rapid vasoconstrictive effect of aldosterone at physiological concentrations. Gunaruwan et al. [23] reported that aldosterone had no acute effect on forearm resistance vessels in healthy male volunteers, which was clearly different from results reported by Romagni et al. and Schmidt et al., who also in turn demonstrated contrary effects of aldosterone on the vascular wall [24, 25]. These inconsistent in vivo study results appear to raise more questions concerning how aldosterone acts on BP regulation than they have attempted to answer.

Some possible mechanisms through which secondary hypertension may modify the circadian BP include sympathetic nervous system activation, fluid retention, and increases in peripheral vascular resistance. Inappropriate activation of the sympathetic nervous system during sleep has been considered to be a major factor contributing to elevation of sleep BP in pheochromocytoma [26], hyperthyroidism [26], and sleep apnea [27]. However, this is not the case in primary aldosteronism [28]. Uzu et al. indicated that sodium restriction shifted the circadian BP rhythm from “nondipper” to “dipper” status in salt-sensitive patients with EH [29] and restored the nocturnal BP dip in those with APA [30].

Our study had some limitations. The sample size was relatively small. Within the 24-h in-hospital ABPM, blood samples for measurements of plasma aldosterone were drawn at midnight, 0400, 0800, 1200, 1600, and 2000 h; and blood sampling and hospitalization may have impaired sleeping patterns and the nocturnal dip of BP. This could have had a significant effect on the results. Moreover, we only measured seven time points for plasma aldosterone, and it is possible that more frequent blood sampling for aldosterone measurement would have offered a more precise estimation of the aldosterone rhythm. We used 24-h urine vanillylmandelic acid to exclude pheochromocytoma; however, this is not the gold standard method for the diagnosis of pheochromocytoma. These major limitations are likely to have weakened the strength of our research and should be taken into consideration when analyzing or applying the findings of our study.

Studies with larger samples and with more frequent blood sampling for aldosterone measurement or studies that use another technique that is not affected due to hospitalization are warranted to further evaluate the circadian BP profile in patients with APA and its relationship with circadian aldosterone rhythm.

In conclusion, the circadian BP pattern was not associated with a change in PAC in patients with APA. Further studies are needed to address the mechanisms involved in the reduction of nocturnal BP decline in patients with APA.

## Figures and Tables

**Table 1:** Demographic and biochemical values in patients with aldosterone-producing adenoma and essential hypertension.

	APA (n = 27)	EH (n = 27)	P-value
Age (years)	45.9 ± 11.0	41.6 ± 12.4	0.188
Sex (male/female)	13/14	13/14	1.000
Body mass index (kg/m^2^)	23.7 ± 3.7	24.5 ± 3.6	0.407
Duration of hypertension (weeks)^*∗*^	153 (58, 330)	156 (46,306)	0.945
Family history of hypertension (yes/no)	15/12	14/13	1.000
Serum potassium (mmol/L)	2.62 ± 0.71	3.62 ± 0.55	<0.001
Serum total cholesterol (mmol/L)	4.64 ± 1.09	4.88 ± 1.12	0.450
Serum triglyceride (mmol/L)	1.25 ± 0.57	1.43 ± 1.32	0.518
Serum HDL-c (mmol/L)	1.24 ± 0.29	1.15 ± 0.25	0.200
Serum LDL-c (mmol/L)	3.02 ± 0.97	3.27 ± 0.99	0.368
Basal PAC (ng/dL)	21.8 ± 6.3	18.1 ± 6.5	0.049
Post challenge PAC (ng/dL)	23.5 ± 7.2	18.5 ± 4.6	0.006
Basal PRA (ng/mL/h)^*∗*^	0.050 (0.040, 0.308)	1.665 (1.052, 3.247)	<0.001
Post challenge PRA (ng/mL/h)^*∗*^	0.058 (0.052, 0.326)	6.887 (4.587, 10.86)	<0.001
Basal ARR^*∗*^	306 (240, 574)	12.9 (8.7, 35.9)	<0.001
Post challenge ARR^*∗*^	339 (219, 670)	2.89 (1.64, 6.75)	<0.001
24-h urine aldosterone (ug/24-hour)	4.92 ± 2.69	3.1 1 ± 1.84	0.019

^*∗*^not normally distributed, data expressed as median (25^th^ percentile, 75^th^ percentile)

APA, aldosterone-producing adenoma; EH, essential hypertension; PAC, plasma aldosterone concentration; PRA, plasma renin activity; ARR, aldosterone renin ratio, calculated as PAC (ng/dL)/PRA (ng/mL/h)

**Table 2:** Comparison of blood pressure measurements between patients with aldosterone-producing adenoma and essential hypertension.

	APA (n = 27)	EH (n = 27)	P value
Mean BP (mm Hg)			
day time SBP	143.8 ± 13.3	137.9 ± 18.5	0.188
day time DBP	94.2 ± 10.4	89.4 ± 15.5	0.189
night time SBP	137.8 ± 15.0	130.3 ± 19.6	0.120
night time DBP	87.9 ± 11.9	83.0 ± 17.7	0.242
Day-night BP difference (mm Hg)			
SBP	5.85 ± 9.40	7.63 ± 11.20	0.530
DBP	6.41 ± 6.34	6.30 ± 6.78	0.951
Relative night time decline (%)			
SBP	4.01 ± 6.52	5.38 ± 7.85	0.490
DBP	6.84 ± 6.91	7.24 ± 8.21	0.845
BP pattern (dipper/nondipper)^*∗*^	5/22	7/20	0.513

^*∗*^dippers were defined as those with a >10% decline in both systolic and diastolic blood pressure

Note: day-night BP difference calculated as day time BP minus night time BP, and relative night time decline calculated as: (day time BP – night time BP) ÷ day time BP × 100%

APA, aldosterone-producing adenoma; BP, blood pressure; DBP, diastolic blood pressure; EH, essential hypertension; SBP, systolic blood pressure

**Table 3:** Linear correlation of plasma aldosterone concentration and blood pressure.

	APA				EH			
	SBP^*∗*^	SBP1h^*∗∗*^	DBP^*∗*^	DBP1h^*∗∗*^	SBP^*∗*^	SBP1h^*∗∗*^	DBP^*∗*^	DBP1h^*∗∗*^
R	0.306	0.471	0.446	0.390	0.559	0.539	0.611	0.742
P	0.556	0.345	0.375	0.444	0.248	0.270	0.198	0.091

^*∗*^BP recorded at the time of blood sampling for aldosterone measurement

^*∗∗*^BP recorded 1 h after blood sampling for aldosterone measurement

APA, patients with aldosterone-producing adenoma; BP, blood pressure; DBP, diastolic blood pressure; EH, patients with essential hypertension; SBP, systolic blood pressure

## Data Availability

The data used to support the findings of this study are available from the corresponding author upon request.

## Abbreviations

APA: Aldosterone-producing adenoma
BP: Blood pressure
DBP: Diastolic blood pressure
EH: Essential hypertension
PA: Primary aldosteronism
PAC: Plasma aldosterone concentration
PRA: Plasma renin activity
SBP: Systolic blood pressure.
